# From belief to behavior: behavioral engagement as the proximal link between self-efficacy and L2 achievement

**DOI:** 10.3389/fpsyg.2026.1997825

**Published:** 2026-10-19

**Authors:** Guoqiang Zhang, Fu an Zhang, Yong Li, Yanping Gong, Fang He

**Affiliations:** 1 Jiyang College of Zhejiang A&F University, Zhuji, China; 2 Zhejiang A&F University, Hangzhou, China

**Keywords:** behavioral engagement, L2 achievement, learning engagement, self-efficacy, sequential mediation

## Abstract

**Background:**

Learning engagement (LE) has been recognized as a key mediator connecting learning beliefs and achievement, but previous studies have examined its sub-dimensions—behavioral (BE), emotional (EE), and cognitive (CE) engagement—either in isolation or as parallel constructs, overlooking their potential sequential relationships. This analytical approach may obscure how these dimensions work together and yield inconsistent findings.

**Method:**

Drawing on Social Cognitive Theory (SCT) and the Broaden-and-Build Theory (BBT), this study employed structural equation modeling (SEM) to compare a sequential mediation model with a parallel alternative among Chinese EFL undergraduates preparing for the CET-4. Self-efficacy and the three engagement dimensions were measured concurrently approximately two months before CET-4, with its scores obtained from official records upon release.

**Results:**

The sequential model fit the data better than the parallel alternative, consistent with the hypothesized sequential framework. Self-efficacy was associated with BE through independent emotional and cognitive pathways, and a serial pathway with EE theoretically specified prior to CE. Among the engagement dimensions, only BE was directly associated with subsequent L2 achievement. Self-efficacy also retained a direct association with achievement.

**Conclusion:**

Within the hypothesized engagement framework, BE was the most proximal engagement dimension associated with internal psychological states and L2 achievement. The findings offer an integrative perspective on past inconsistencies and suggest that self-efficacy is associated with language test performance through both sustained behavioral investment and direct, unmediated processes.

## 1 Introduction

In L2 acquisition research, understanding how learners’ psychological beliefs, particularly self-efficacy, translate into academic achievement remains a significant concern. Self-efficacy is a consistent predictor of L2 achievement (Goetze and Driver, 2022; Wang et al., 2021), as it provides learners with the motivational drive and resilience needed for complex language tasks. The literature has documented that efficacy beliefs often affect academic outcomes through learning engagement (LE) (Cai and Xing, 2023; Huang and Liu, 2025; Luo et al., 2023).

Nevertheless, when investigating the mediating role of LE in connecting self-efficacy and L2 achievement, previous research has generally treated LE as a monolithic construct, rarely examining the specific roles of its sub-dimensions (Feng and Hong, 2022; Wang et al., 2023). Even when these dimensions are occasionally explored (Cai and Xing, 2023; Reeve et al., 2025), the existing literature remains fragmented, yielding inconsistent conclusions. For instance, some studies identify emotional engagement (EE) as a critical bridge linking self-efficacy to academic achievement (Song and Wang, 2026; Xu, 2024), whereas others conclude that among the three LE dimensions, only cognitive engagement (CE) mediates this relationship (Cai and Xing, 2023). These diverging conclusions may stem from how LE is modeled: existing research tends to either examine a single dimension in isolation, or treat the three sub-dimensions as parallel, independent constructs. Such isolated or parallel approaches tend to overlook the dynamic, sequential interactions among engagement dimensions (Hong et al., 2020; Philp and Duchesne, 2016). This oversight is particularly problematic in the test-oriented Chinese EFL context, where intense academic pressure often triggers a critical disconnect between learners’ internal psychological engagement (e.g., emotional states and cognitive planning) and their actual behavioral output.

To address this limitation, the present study adopts a sequential mediation perspective grounded in Social Cognitive Theory (SCT; Bandura, 1986) and Broaden-and-Build Theory (BBT; Fredrickson, 1998, 2001). Specifically, SCT posits that self-efficacy regulates human functioning through internal affective (EE) and cognitive states (CE), which are then expressed in behavioral practice (BE) to facilitate actual outcomes (Bandura, 1986). Complementing this, BBT proposes that positive affective experiences (EE) broaden learners’ cognitive scope, thereby facilitating deeper cognitive planning (CE) (Fredrickson, 1998). Integrating these frameworks, we specify a theory-guided sequential model in which EE and CE are modeled as antecedents of BE, with EE theorized to precede CE. Within this model, BE is specified as the most proximal engagement dimension linking internal psychological readiness to L2 achievement. Building on this framework, we constructed a multiple-pathway model to examine these hypothesized pathways among Chinese EFL learners.

The present study aims to: (1) examine whether the data are consistent with the proposed sequential structure of LE dimensions; (2) test the hypothesized bridging role of BE in linking psychological beliefs to subsequent achievement; and (3) examine the direct and indirect pathways associating self-efficacy with L2 achievement. By testing this integrated, theory-guided model, we seek to clarify the pattern of associations among engagement dimensions and inform directions for future instructional research in EFL contexts.

## 2 Literature review

### 2.1 The predictive role of self-efficacy in L2 achievement

Self-efficacy, a central construct of Bandura’s Social Cognitive Theory, refers to individuals’ belief in their capability to execute actions to handle specific tasks (Bandura, 1986, 1993). As this framework posits, it underpins human agency, shaping how individuals feel, think, and behave in the face of challenges. In the L2 learning domain, this construct is contextualized as learners’ perceived competence in mastering specific linguistic skills and navigating communicative tasks (Cancino et al., 2022; Goetze and Driver, 2022; Kim, 2024). Here, it operates as a proactive psychological drive. Specifically, it influences learners’ willingness to engage with complex language tasks and their resilience when encountering linguistic obstacles (Fathi et al., 2024; Phipps, 2022; Teravainen-Goff, 2025).

Self-efficacy has consistently been identified as a strong predictor of L2 achievement (Cai and Xing, 2023; Kim and Shin, 2021; Kutuk et al., 2023; Wang and Sun, 2020; Wang and Wang, 2026). This effect is partly explained by the ways learners engage with language tasks, as learners with strong efficacy beliefs tend to exhibit deeper cognitive processing, sustained effort, and more positive emotional responses (Bai and Wang, 2023; Cai and Xing, 2023; Goetze and Driver, 2022; Kim, 2024; Wang et al., 2025; Wang et al., 2021). Moreover, this predictive relationship has been documented in diverse educational settings, from primary classrooms (Bai et al., 2020; Bai and Wang, 2023) to university-level EFL instruction (Jiang, 2025; Lu et al., 2022). The relationship also extends beyond overall proficiency to specific language skills: writing self-efficacy predicts writing performance (Prat-Sala and Redford, 2012; Xu W. et al., 2023), speaking self-efficacy predicts oral competence (Mega et al., 2026; Pramerta, 2021), and similar patterns hold for reading (Jiang, 2023; Unrau et al., 2018).

While the above evidence robustly links self-efficacy to L2 achievement, these findings predominantly stem from communicative, skill-building environments (Bai et al., 2020; Jiang, 2023; Pramerta, 2021; Prat-Sala and Redford, 2012; Unrau et al., 2018). How this relationship operates under high-stakes, test-oriented conditions, such as the College English Test Band 4 (CET-4), has received far less attention (Cai and Zhao, 2023; Kutuk et al., 2023). In such a competitive ecosystem, L2 achievement is largely measured by performance on standardized, time-pressured tests (Bayliss and Raymond, 2004; Brown et al., 2018; Ihlenfeldt and Rios, 2022), where success depends as much on test-taking skill as on English ability. Although self-efficacy exerts some direct influence on test performance, its influence on achievement is also likely to operate through self-regulatory processes. Highly efficacious learners are more likely to proactively seek out effective test-taking strategies, persist in practice when facing challenges (Teng, 2021; Üner et al., 2020), and maintain positive emotional engagement under high-stakes pressure (Huang and Liu, 2025). Thus, in test-oriented contexts, the critical question is not whether self-efficacy matters, but how it matters: understanding the mechanisms through which these beliefs translate into actual test performance is essential for a complete account of the efficacy-achievement link. LE represents one such mechanism, increasingly recognized as a promising mediator (Hao and Lu, 2024; Kim, 2024; Luo et al., 2023).

Taken together, these studies establish a robust self-efficacy-achievement association but leave three issues unresolved: most rely on communicative or course-based outcomes rather than high-stakes standardized test scores; many treat engagement as a global construct; and few specify how engagement dimensions are related to one another (Cai and Xing, 2023; Liu M. et al., 2023). The present study therefore addresses not only which dimension(s) mediate the association, but also how engagement dimensions are interrelated in a test-oriented EFL context.

### 2.2 The dilemma in learning engagement research

To unpack the interplay among these subdimensions, it is first necessary to clarify how LE is structured. LE is conceptualized as a multidimensional construct encompassing three dimensions: behavioral engagement (BE), emotional engagement (EE), and cognitive engagement (CE) (Fredricks et al., 2004; J. Li and Xue, 2023; Wong and Liem, 2022). Within this framework, BE denotes observable behaviors such as participation, effort, and persistence in learning activities (Reeve et al., 2025; Skinner and Belmont, 1993). EE refers to students’ positive affective responses (e.g., interest and enjoyment) toward learning activities (Meyer and Turner, 2002; Xu X. et al., 2023). Finally, CE reflects the intentional use of deep processing strategies and sustained mental effort (Fredricks et al., 2004; Philp and Duchesne, 2016). However, despite its theoretical multidimensionality, LE has frequently been treated as a monolithic construct in empirical research (Hao and Lu, 2024; Kim, 2024).

While evidence suggests that LE as a holistic construct mediates the relationship between self-efficacy and L2 achievement (Hao and Lu, 2024; Kim, 2024; Luo et al., 2023), the specific mediating roles of individual sub-dimensions remain underexplored. Cai and Xing (2023) disaggregated LE into its three dimensions and tested them as parallel mediators between self-efficacy and language achievement, finding that only CE produced a significant indirect effect. This finding is consistent with evidence that cognitive strategies and self-regulation are robustly linked to L2 achievement (Jia, 2022; Liu Q. et al., 2023; Meng and Zhang, 2023). Yet this apparent primacy may reflect analytical constraints rather than theoretical reality. When examined in isolation, BE has been shown to mediate the link between emotional variables and achievement (Feng and Hong, 2022; Kang and Wu, 2022). A similar inconsistency characterizes EE: it predicts CET-4 scores more strongly than the other two dimensions (Liu M. et al., 2023), yet He et al. (2024) found that it did not mediate between L2 grit and academic achievement. These divergent findings suggest that the predictive power of each engagement dimension depends on how it is modeled.

While studies examining individual dimensions have advanced our understanding, the field has developed a methodological blind spot. Prior studies have typically adopted one of two strategies: isolating a single dimension to establish its standalone effect (Feng and Hong, 2022; Kang and Wu, 2022) or entering all three simultaneously as competing predictors (Liu M. et al., 2023) or parallel mediators (Cai and Xing, 2023). Both strategies treat EE, BE, and CE as if they were independent of one another, although there is considerable evidence that the three dimensions overlap and are closely related (Estévez et al., 2021; Philp and Duchesne, 2016). When one dimension is examined alone, its effect may partly include the variance it shares with the other two. When all three are examined as parallel mediators, their shared variance is partitioned across the mediators, and this may mask paths that are significant when examined alone. In both cases, a dynamic motivational-behavioral process is reduced to a static snapshot. Consequently, the dominance of CE observed in parallel mediator models may partly reflect these analytical constraints rather than a theoretical truth. This synthesis indicates that the central problem is not simply whether engagement mediates the self-efficacy-achievement link, but how the dimensions are jointly modeled. The present study responds by testing such a structure, while explicitly acknowledging that concurrently measured self-reports cannot establish temporal ordering.

To address this blind spot, the present study specifies the three engagement dimensions as a theory-guided sequential structure rather than as concurrent competitors. The theoretical rationale is developed below.

### 2.3 From beliefs to actions: a multi-path sequential framework

To make sense of these mixed findings, this study draws on two theoretical perspectives: Social Cognitive Theory (SCT; Bandura, 1986) and the Broaden-and-Build Theory (BBT; Fredrickson, 2001). The present study does not seek to determine which single dimension dominates, but to examine whether self-efficacy is associated with these dimensions through hypothesized ordered pathways and whether these pathways are linked to L2 achievement.

SCT posits that self-efficacy shapes performance through affective, cognitive, and behavioral pathways (Bandura, 1986). In this framework, affective and cognitive processes serve as internal regulatory mechanisms that prepare and direct behavior, but the translation of beliefs into performance is realized through behavioral activation (Bandura, 1991). The three dimensions of LE offer natural empirical counterparts to these routes: EE captures the affective process, CE the cognitive process, and BE the behavioral process. On this mapping, EE and CE are modeled as the psychological states through which efficacy beliefs are registered, whereas BE is modeled as the dimension through which these states are connected with academic outcomes.

This theory-guided specification reflects both theoretical and contextual considerations. Theoretically, SCT treats behavior as the external expression of internal beliefs (Bandura, 1991, 2004; Beauchamp et al., 2019): efficacy beliefs shape what individuals feel and think, but they produce achievement gains primarily by determining what individuals do (Komarraju and Nadler, 2013; Trautner and Schwinger, 2020; Zimmerman, 2000). Contextually, the CET-4 is a formal, time-bound examination in which score improvements depend on quantifiable behavioral investments—repeated practice with test formats, cumulative vocabulary study, and consistent participation in preparatory activities (Xie, 2013; Xu, 2021; Yan and Huizhong, 2006). Unlike immersion contexts, where spontaneous language use may allow cognitive and emotional gains to directly influence performance (Faretta-Stutenberg and Morgan-Short, 2018; Sun et al., 2024), the CET-4 rewards accumulated, deliberate practice. In this setting, internal resources are less likely to translate into score gains without sustained behavioral effort. BE is therefore modeled as the proximal link to L2 achievement.

These considerations suggest two hypothesized pathways linking self-efficacy to BE. First, efficacy beliefs are expected to be associated with adaptive emotional states that are also linked to the effort and persistence characteristic of BE. Second, efficacy beliefs are expected to be associated with deep processing and self-regulatory strategies that may be expressed in active learning behaviors. In both cases, these pathways converge on BE.

While SCT suggests that emotional and cognitive states are expressed through behavior, it does not specify their directional relationship. The broaden-and-build theory addresses this by proposing that positive emotions expand thought-action repertoires and build enduring cognitive resources (Fredrickson, 1998, 2001). In educational contexts, this theoretical proposition has been linked to deeper cognitive engagement and strategic processing (Philp and Duchesne, 2016). Applied to the present framework, this motivates a third hypothesized pathway in which self-efficacy is associated with positive affect, positive affect is modeled as an antecedent of cognitive processing, and both are linked to BE. In applying BBT, however, we treat EE as a relatively generalized affective orientation toward English learning rather than as a momentary positive emotional state. This treatment is defensible on two grounds. On the measurement side, habitual and fleeting emotional states are distinct yet equally legitimate objects of study (Pekrun, 2006); on the functional side, a stable positive disposition broadens thinking and supports flexible coping much as momentary emotions do (Fredrickson and Joiner, 2002). Accordingly, BBT serves as a theoretical heuristic for specifying the EE-to-CE path. In high-stakes EFL examinations where performance pressure is acute, learners may alternatively deploy cognitive strategies before positive emotions develop (Bielak and Mystkowska-Wiertelak, 2024; Zhang and Wang, 2024). Because EE and CE were measured concurrently, their ordering cannot be definitively settled. The present study therefore specifies the hypothesized EE-to-CE sequence on theoretical grounds, drawing on BBT, while acknowledging the cognitive-first alternative as a viable competing account that awaits longitudinal confirmation.

The preceding discussion points to three hypothesized indirect pathways linking self-efficacy to L2 achievement: an affective pathway (SE → EE → BE), a cognitive pathway (SE → CE → BE), and a serial pathway that combines the two (SE → EE → CE → BE). In each pathway, BE constitutes the final link to L2 achievement. Guided by this framework, the study proposes the following hypotheses:

*H1*: Self-efficacy is indirectly associated with BE through EE and CE (SE → EE → BE; SE → CE → BE).

*H2*: The indirect association between self-efficacy and BE can be decomposed into independent and sequential pathways involving EE and CE:

*H2a*: Self-efficacy is indirectly associated with BE through EE (SE → EE → BE).

*H2b*: Self-efficacy is indirectly associated with BE through CE (SE → CE → BE).

*H2c*: Self-efficacy is indirectly associated with BE through a sequential pathway specifying the EE → CE ordering (SE → EE → CE → BE).

*H3*: BE is positively associated with subsequent L2 academic achievement (BE → L2 achievement).

*H4*: Self-efficacy retains a direct positive association with L2 achievement beyond the indirect pathways (SE → L2 achievement).

To test these hypotheses, a two-wave time-lagged design with SEM was employed. The following section details the participants, instruments, and analytical procedures (Figure 1).

**Figure 1:**
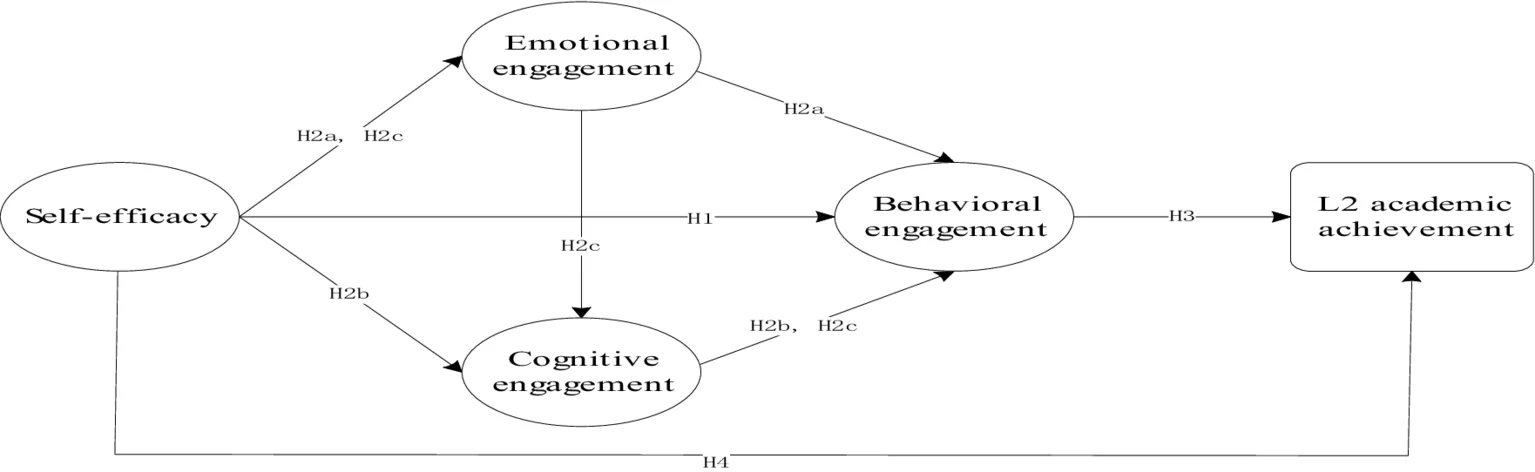
Hypothetical model of the mediating role of learning engagement sub-dimensions. H = hypothesis; H1 proposed that the effect of self-efficacy on behavioral engagement is mediated by emotional and cognitive engagement (see H2a-H2c for specific pathways).

## 3 Method

### 3.1 Participants

Participants were sophomore undergraduates drawn from three comprehensive universities in Zhejiang Province, China. This cohort represents the target population for the present research question: students at the stage where mandatory foundational English instruction has just concluded and high-stakes standardized testing is imminent. This demographic was selected for two reasons. First, as sophomores scheduled to take the National College English Test Band 4 (CET-4) in December 2024, their academic calendar offered an ideal time-lagged design in which self-efficacy and engagement could be measured prior to the examination and subsequent achievement captured through standardized test scores. Second, having recently completed the same mandatory freshman English curriculum, they shared a homogeneous instructional background, reducing variability attributable to differential freshman English coursework.

Data were collected in two waves. A total of 673 questionnaires were collected. To ensure data quality, three exclusion criteria were applied: (1) unfeasibly short completion times (less than 100 s); (2) straight-lining response patterns (e.g., choosing the same option for all items); and (3) missing data on key variables. After screening, 650 valid responses were retained (effective response rate = 96.6%). The final sample comprised 232 males (35.7%) and 418 females (64.3%), with a mean age of 18.4 years (*SD* = 0.71). All participants were native Chinese speakers studying English as a foreign language (EFL).

Ethical considerations. This study was conducted in accordance with the Declaration of Helsinki and received ethical approval from the Ethics Committee of Jiyang College of Zhejiang A&F University (Approval No. 02). All participants provided digital informed consent prior to completing the survey, acknowledging the study purpose, data confidentiality, and their right to withdraw at any time.

### 3.2 Procedure

Data collection employed a two-wave time-lagged design. At Time 1 (T1; October–November 2024), self-report measures of psychological states and engagement were administered online via Wenjuanxing, a widely used Chinese survey platform. To facilitate subsequent data matching, participants were asked to provide their student IDs, with explicit assurance that these identifiers would be used exclusively for data linkage and would remain confidential.

At Time 2 (T2; February 2025, following the release of the December 2024 CET-4 results), participants’ objective test scores were collected and matched with their T1 responses. By temporally separating self-reported psychological variables (T1) from objective test scores (T2), this design reduces the risk of common method bias. It should be noted, however, that because all self-report predictors were measured concurrently at T1, temporal separation applies to the predictors and outcome but not to the ordering among self-efficacy, EE, CE, and BE. Accordingly, mediation among the T1 variables remains cross-sectional.

### 3.3 Measures

Unless otherwise noted, all self-report scales underwent the same adaptation protocol. First, source items were contextualized to the EFL setting by rewording class-specific references (e.g., “this class,” “the college English class”) as “English learning.” Second, items were translated into Chinese following Brislin’s (1970) translation-back-translation procedure and reviewed by two experts in applied linguistics for content validity. Finally, the questionnaire was pilot-tested with 73 undergraduates from two classes whose responses were not included in the main analyses to check item clarity and completion time; no substantive difficulties were reported. Scale-specific details are reported below.

Self-Efficacy. Self-efficacy was assessed using a 7-item scale adapted from the self-efficacy subscale of the Motivated Strategies for Learning Questionnaire (Pintrich and De Groot, 1990). Of the nine original items, two were excluded: one tapping expected grades rather than capability beliefs, and one tapping perceived subject knowledge, which would conflate self-efficacy with prior knowledge (see Supplementary Table S5 for the complete item). Participants responded on a 5-point Likert scale ranging from 1 (strongly disagree) to 5 (strongly agree). A sample item is “I’m certain I can understand the difficult material in English learning.” Confirmatory factor analysis (CFA) indicated adequate fit for the unidimensional model: *χ^2^* = 54.85, *df* = 14, *CFI* = 0.973, *TLI* = 0.960, *RMSEA* = 0.064 [90% *CI* = 0.045, 0.083], *SRMR* = 0.027. The scale demonstrated high internal consistency (Cronbach’s *α* = 0.90). Factor loadings, composite reliability (*CR* = 0.901), and average variance extracted (*AVE* = 0.566) are reported in Supplementary Table S1.

Learning engagement. Learning engagement was measured with a 25-item scale adapted from the Foreign Language Classroom Engagement Scale (Guo et al., 2022). The source scale comprises 38 items across eight dimensions; for the present study, 25 items were selected and reorganized by engagement type to align with the three-dimensional framework of Fredricks et al. (2004). Item selection followed three criteria: (a) content alignment with the target engagement dimension, (b) standardized loadings above 0.60 on the source scale (Guadagnoli and Velicer, 1988), and (c) the absence of semantic redundancy and construct contamination across dimensions. The complete item list and item-source are provided in Supplementary Table S6. The scale comprises BE (8 items; e.g., “In English learning, I listen very carefully.”), EE (8 items; e.g., “I feel happy communicating and interacting with my classmates in English learning.”), and CE (9 items; e.g., “In English learning, I relate what I’m learning to what I already know.”). Participants responded on the same 5-point Likert scale. The BE items capture perceived rather than observed behavioral engagement, and the EE items capture a generalized affective orientation rather than momentary emotional states. CFA results supported the three-factor structure: *χ^2^* = 942.83, *df* = 267, *CFI* = 0.919, *TLI* = 0.909, *RMSEA* = 0.062 [90% *CI* = 0.058, 0.067], *SRMR* = 0.068. All sub-dimensions exceeded the *CR* > 0.70 threshold; EE and CE exceeded *AVE* > 0.50 threshold, and the marginal BE value (*AVE* = 0.494) was deemed acceptable given its strong reliability (*CR* = 0.885) and theoretical distinctiveness (Bagozzi and Yi, 1988; Fornell and Larcker, 1981). HTMT ratios ranged from 0.363 to 0.848, all below the recommended 0.85 threshold (Henseler et al., 2015) and Fornell-Larcker comparisons supported discriminant validity (Fornell and Larcker, 1981). Internal consistency was high for the overall scale (*α* = 0.95) and its sub-dimensions (BE: *α* = 0.88; EE: *α* = 0.92; CE: *α* = 0.93). Factor loadings, CR, and AVE values are reported in Supplementary Table S1.

L2 achievement. L2 achievement was operationalized as participants’ total scores on the December 2024 CET-4. Administered by the Chinese Ministry of Education, the CET-4 is a national standardized test with a maximum score of 710, comprising listening (35%), reading (35%), writing (15%), and translation (15%) sections. As a high-stakes examination widely used in Chinese higher education, it is widely regarded as a reliable indicator of college students’ comprehensive English proficiency. In the present sample, the mean CET-4 score was 442 (*SD* = 92), slightly above the passing threshold of 425.

### 3.4 Data analysis

Data analysis proceeded in three stages. First, correlational analyses and Harman’s single-factor test were conducted using SPSS 27.0 as a preliminary check for common method bias. Second, the measurement model was validated via CFA in Mplus 8.3 using robust maximum likelihood (MLR) with all constructs specified simultaneously. Third, SEM was performed using MLR to account for potential non-normality in the Likert-scale indicators. Model fit for both CFA and SEM was evaluated against standard thresholds: CFI > 0.90, TLI > 0.90, RMSEA < 0.08, and SRMR < 0.08.

Within the SEM stage, two analytical steps were carried out. In the first step (model comparison), the multiple-pathway sequential model (Model 1) was compared against the traditional parallel mediation model (Model 2), in which the structural paths linking the engagement sub-dimensions were constrained to zero (i.e., EE → BE, CE → BE, and EE → CE). Because Model 2 was nested within Model 1, model fit was compared using the Satorra-Bentler scaled chi-square difference test, supplemented by AIC and BIC. To evaluate the robustness of the EE-to-CE specification, two non-nested alternatives were also estimated with MLR: a CE-first model (Model 3), in which the CE → EE ordering was specified, and a reciprocal model (Model 4), in which bidirectional paths between EE and CE were allowed. Given that EE and CE were measured concurrently, these comparisons were used to assess statistical compatibility rather than to establish temporal order. In the second step (mediation testing), because Mplus does not permit bootstrapping in conjunction with MLR, the model retained from the first step was re-estimated under maximum likelihood (ML) with bias-corrected percentile bootstrapping (5,000 resamples); the 95% confidence intervals for direct and indirect effects reported in Table 1 were obtained from this bootstrap re-estimation rather than from the MLR-based model comparison. The CE-first alternative (Model 3) was likewise re-estimated under this procedure for the comparison of indirect effects. The direct path from self-efficacy to L2 achievement was retained throughout to assess whether self-efficacy showed a significant association independent of the engagement mediators.

**Table 1:** Sequential mediation pathways of self-efficacy to behavioral engagement and L2 achievement.

Paths	Mediating effects	Effect	SE	Est/SE	*p*	Bootstrap 95% CI	
						LLCI	ULCI
SE → BE	Total Effect	0.333	0.039	8.440	< 0.001	0.255	0.410
	Direct Effect	−0.026	0.049	−0.527	0.598	−0.122	0.071
	Indirect Effect	0.359	0.033	10.924	< 0.001	0.294	0.423
	Ind1: SE → EE → BE	0.194	0.047	4.158	< 0.001	0.102	0.285
	Ind2: SE → CE → BE	0.038	0.014	2.640	0.008	0.010	0.066
	Ind3: SE → EE → CE → BE	0.127	0.034	3.777	< 0.001	0.061	0.193
SE → LA	Total Effect	0.239	0.039	6.121	< 0.001	0.162	0.315
	Direct Effect	0.131	0.052	2.536	0.011	0.030	0.232
	Indirect Effect	0.108	0.032	3.350	0.001	0.045	0.171
	Inds through BE:						
	Ind4: SE → BE → LA	−0.005	0.010	−0.522	0.602	−0.025	0.015
	Ind5: SE → EE → BE → LA	0.040	0.013	2.934	0.003	0.013	0.066
	Ind6: SE → CE → BE → LA	0.008	0.003	2.227	0.026	0.001	0.015
	Ind7: SE → EE → CE → BE → LA	0.026	0.009	2.784	0.005	0.008	0.044
	Inds bypassing BE:						
	Ind8: SE → EE → LA	0.028	0.049	0.575	0.565	−0.068	0.124
	Ind9: SE → CE → LA	0.003	0.010	0.259	0.795	−0.018	0.023
	Ind10: SE → EE → CE → LA	0.009	0.035	0.260	0.795	−0.060	0.078

Ind = indirect effect; SE = self-efficacy; BE = behavioral engagement; EE = emotional engagement; CE = cognitive engagement; LA = L2 achievement; bootstrap sample size = 5,000; LLCI = lower limit of the confidence interval; ULCI = upper limit of the confidence interval.

## 4 Results

### 4.1 Common method bias test

The two-wave time-lagged design, together with an objective outcome measure (CET-4 scores), reduces the risk of common method bias (CMB). As an additional check, an unrotated principal component analysis using Harman’s single-factor test was conducted on the T1 self-report items. Six factors with eigenvalues greater than 1 were extracted, with the first principal factor accounting for 42.01% of the total variance—well below the commonly cited threshold of 50% (Podsakoff and Organ, 1986). Taken together, these procedural and statistical indicators suggest that CMB is unlikely to be a serious concern in this study.

### 4.2 Bivariate correlations

Pearson correlation coefficients for all variables are presented in Table 2. Self-efficacy was significantly and positively correlated with all three sub-dimensions of LE (BE, EE, and CE), with coefficients ranging from 0.333 to 0.600 (*p* < 0.01). Of these three, BE exhibited the strongest positive correlation with L2 achievement (*r* = 0.285, *p* < 0.01), followed by EE (*r* = 0.252, *p* < 0.01) and CE (*r* = 0.239, *p* < 0.01). Self-efficacy also demonstrated a significant positive correlation with academic achievement (*r* = 0.239, *p* < 0.01). These bivariate associations provide a basis for testing the structural model.

**Table 2:** Correlation matrix of variables.

Variable	Self-efficacy	Behavioral engagement	Emotional engagement	Cognitive engagement
Behavioral engagement	0.333^**^			
Emotional engagement	0.600^**^	0.547^**^		
Cognitive engagement	0.575^**^	0.538^**^	0.623^**^	
L2 achievement	0.239^**^	0.285^**^	0.252^**^	0.239^**^

^**^p < 0.01.

### 4.3 Sequential mediation analysis

#### 4.3.1 Model comparison

Prior to interpreting path coefficients, the hypothesized sequential model (Model 1) was compared against the traditional parallel mediation model (Model 2). Model 1 exhibited acceptable fit to the data (*χ^2^* = 1247.406, *df* = 482, *CFI* = 0.929, *TLI* = 0.922, *RMSEA* = 0.049 [90% *CI* = 0.046, 0.053], *SRMR* = 0.061). In contrast, Model 2 exhibited relatively poor fit (*χ^2^ = 1617.424*, *df* = 485, *CFI* = 0.895, *TLI* = 0.886, *RMSEA* = 0.060 [90% *CI* = 0.057, 0.063], *SRMR* = 0.136). The Satorra-Bentler scaled chi-square difference test indicated a significant deterioration in fit (*Δχ^2^* = 352.066, *Δdf* = 3, *p* < 0.001), and Model 2 produced markedly higher AIC and BIC values relative to Model 1 (*ΔAIC* = 450.592, *ΔBIC* = 437.161). This indicates that treating the three engagement dimensions as independent parallel mediators (without the hypothesized linkages among them) does not adequately capture their structural relationships.

To evaluate the robustness of the EE-to-CE specification, two alternative specifications were then examined. The CE-first model (Model 3) showed fit identical to Model 1 (*χ^2^* = 1247.406, *df* = 482, *CFI* = 0.929, *TLI* = 0.922, *RMSEA* = 0.049 [90% CI = 0.046, 0.053], *SRMR* = 0.061), indicating that the EE-first and CE-first specifications are statistically equivalent given the concurrent measurement of EE and CE at T1. Moreover, when Model 3 was re-estimated under ML with bias-corrected bootstrapping (5,000 resamples) for mediation testing, the reversed CE → EE path was significant (*β* = 0.713, *p* < 0.001), and the serial indirect path SE → CE → EE → BE → L2 achievement remained significant (*β* = 0.027, *p* = 0.004). The reciprocal model (Model 4), in which bidirectional paths between EE and CE were allowed, was not identified with the present single-wave specification, and its fit statistics could therefore not be computed. These results indicate that the data are compatible with both directional orderings and do not empirically establish the EE-to-CE sequence. Model 1 was therefore retained as the focal model for subsequent analyses, with the EE-to-CE ordering justified on theoretical grounds (see Section 2.3) rather than on model fit. Standardized path coefficients and their corresponding significance levels are depicted in Figure 2.

**Figure 2:**
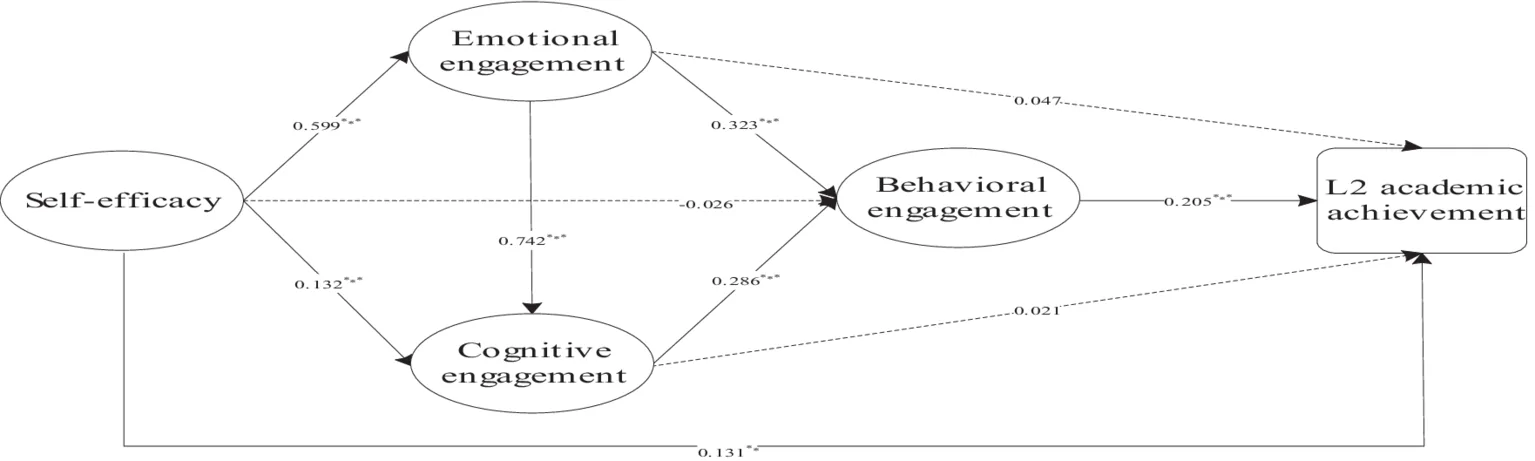
Mediation model with standardized coefficients. solid line: supported hypotheses, dashed line: non-significant paths. *** *p* <. 001, ** *p* <. 05.

#### 4.3.2 Structural paths

Figure 2 presents the standardized path coefficients for the retained model (see Supplementary Table S2 for unstandardized estimates and Supplementary Table S3 for standardized estimates). Self-efficacy was positively associated with both EE (*β* = 0.599, *p* < 0.001) and CE (*β* = 0.132, *p* < 0.001), establishing the prerequisite paths for testing the hypothesized mediation pathways. The direct path from self-efficacy to BE was not significant (*β = −0.026*, *p* = 0.598), indicating that the association between efficacy beliefs and learning behaviors was carried by the internal engagement dimensions. Within the hypothesized engagement structure, EE was positively associated with BE (*β* = 0.323, *p* < 0.001) and CE (*β* = 0.742, *p* < 0.001), while CE was also positively associated with BE (*β* = 0.286, *p* < 0.001). These paths provide the necessary structural basis for the hypothesized independent affective and cognitive routes, as well as the hypothesized EE-to-CE ordering.

Regarding L2 achievement, BE was the only engagement dimension directly associated with L2 achievement (*β* = 0.205, *p* < 0.001). Neither EE (*β* = 0.047, *p = 0*.565) nor CE (*β* = 0.021, *p = 0*.795) showed a significant direct association with L2 achievement, consistent with the view that internal psychological states relate to academic outcomes primarily through behavioral engagement. Meanwhile, the direct association between self-efficacy and L2 achievement remained statistically significant (*β* = 0.131, *p* = 0.011), suggesting that efficacy beliefs retained an independent association with achievement beyond the hypothesized engagement structure.

#### 4.3.3 Mediation effects

The significance of the hypothesized indirect effects was tested using bias-corrected bootstrapping with 5,000 resamples. Standardized estimates, including specific pathways and confidence intervals, are reported in Table 1; nonstandardized estimates are presented in Supplementary Table S4. As shown in Table 1, the total indirect effect of self-efficacy on BE was significant (*β* = 0.359, 95% *CI* [0.294, 0.423]), supporting H1. This total indirect effect was decomposed into three significant specific pathways: the independent emotional route (SE → EE → BE: *β* = 0.194, 95% *CI* [0.102, 0.285]), the independent cognitive route (SE → CE → BE: *β* = 0.038, 95% *CI* [0.010, 0.066]), and the serial route (SE → EE → CE → BE: *β* = 0.127, 95% *CI* [0.061, 0.193]). These results support H2a, H2b, and H2c, respectively.

Regarding L2 achievement, the total indirect effect of self-efficacy was also significant (*β* = 0.108, 95% *CI* [0.045, 0.171]). Decomposing this total indirect effect, all three indirect routes passing through BE reached significance: SE → EE → BE → L2 (*β* = 0.040, 95% *CI* [0.013, 0.066]), SE → CE → BE → L2 (*β* = 0.008, 95% *CI* [0.001, 0.015]), and SE → EE → CE → BE → L2 (*β* = 0.026, 95% *CI* [0.008, 0.044]). In contrast, indirect pathways bypassing BE, namely SE → EE → L2 (*β* = 0.028, 95% *CI* [−0.068, 0.124]), SE → CE → L2 (*β* = 0.003, 95% *CI* [−0.018, 0.023]), and SE → EE → CE → L2 (*β* = 0.009, 95% *CI* [−0.060, 0.078]), were not significant. This pattern indicates that, among the indirect pathways, only those passing through BE reached significance, consistent with the role of BE as the most proximal engagement dimension in the specified model.

In addition, the direct association between self-efficacy and L2 achievement remained significant (*β* = 0.131, *p* = 0.011), supporting H4. Alongside the significant total indirect association, this indicates that self-efficacy showed both direct and indirect associations with L2 achievement. The cumulative indirect effect of the three BE pathways was 0.074, accounting for approximately 31% of the modeled total association between self-efficacy and L2 achievement (*β* = 0.239). This proportion is a descriptive decomposition of the modeled associations, not evidence that 31% of a causal effect has been explained. The remaining portion of the total indirect effect reflects nonsignificant pathways bypassing BE.

## 5 Discussion

This study examined how self-efficacy is associated with L2 achievement through the sub-dimensions of LE among Chinese EFL learners preparing for the CET-4. The results are consistent with a theory-guided sequential mediation framework in which EE, CE, and BE are modeled as a sequential structure rather than as parallel mediators. Self-efficacy was associated with EE and CE, which were likewise associated with BE through independent and sequential pathways. BE was the most proximal engagement dimension linking these internal states to L2 achievement. In addition, self-efficacy remained significantly associated with achievement. These findings carry implications for understanding the role of human agency in high-stakes language learning contexts.

### 5.1 Behavioral engagement and L2 achievement

Our results highlight the central role of BE in the associations between self-efficacy and L2 achievement. Among the three engagement sub-dimensions, only self-reported BE was directly associated with academic outcomes. This indicates that, within the hypothesized sequential structure, EE and CE functioned as distal components indirectly linked to achievement through BE, consistent with the view that self-reported BE constitutes the final link between internal psychological resources and academic outcomes.

This finding is consistent with the core premise of SCT that human agency is expressed through behavior (Bandura, 1986). When direct paths from EE and CE to L2 achievement were tested, neither reached significance. This absence of direct associations suggests that positive affect and deep processing may remain internal resources unless they are reflected in learning behaviors (Bandura, 1991; Fathi et al., 2024; MacIntyre and Gregersen, 2012; Teravainen-Goff, 2025). This observation is also consistent with engagement research (Guo et al., 2022; Tong and Singh, 2025), which positions behavioral action as the critical pedagogical target in language acquisition.

These findings offer an integrative perspective on divergent conclusions from previous studies. While Liu M. et al. (2023) emphasized EE as the strongest predictor of CET-4 performance, and Cai and Xing (2023) identified CE as significant in a parallel mediation framework, these divergent findings may reflect that each study captured a different component of the same hypothesized process. In the present study, the poor fit of the parallel model (Model 2) is consistent with this interpretation: treating engagement sub-dimensions as independent predictors oversimplifies their relationship—a problem compounded by evidence that the three dimensions overlap and interdepend (Estévez et al., 2021; Philp and Duchesne, 2016). Instead, within the specified model, EE and CE were linked to achievement only indirectly through their associations with BE.

In the Chinese EFL context, this behavioral investment carries particular practical weight. High-stakes standardized testing rewards sustained effort—regular practice, vocabulary accumulation, and repeated exposure to test formats—rather than momentary confidence or strategic insight alone (Xie, 2013; Xu, 2021). Consistent with this, our data suggest that EE and CE were linked to test performance primarily through behavioral investment.

### 5.2 Multiple pathways to behavioral engagement

Behavioral engagement was the engagement dimension most directly associated with achievement, but the direct path from self-efficacy to BE was not significant. This pattern is consistent with research indicating that confidence alone does not automatically translate into action (Goetze and Driver, 2022; Teravainen-Goff, 2025): strong confidence may be channeled through specific psychological mechanisms before it is reflected in action. Rather than contradicting Social Cognitive Theory, this pattern aligns with its view that efficacy beliefs are associated with emotional and cognitive resources that may energize and direct behavior (Bandura, 1997). In the Chinese EFL context, where CET-4 preparation demands months of sustained practice (Xie, 2013; Yan and Huizhong, 2006), this gap is particularly consequential—students may feel capable of passing the exam yet fail to maintain routines such as vocabulary practice or mock tests required for score improvement.

Our results identify three such mechanisms. First, the sequential pathway from EE to CE was significant, indicating that positive affect was associated with deeper cognitive processing, which was also associated with behavioral engagement. This aligns with the Broaden-and-Build Theory (Fredrickson, 1998, 2001). The present findings thereby extend this framework to the high-stakes EFL context, where the hypothesized EE-to-CE pathway was linked to sustained behavioral engagement. It is important to note that this hypothesized EE-to-CE ordering was specified on theoretical grounds; the present design does not allow empirical adjudication between this sequence and the cognitive-first alternative.

Second, the independent affective pathway (self-efficacy → EE → BE) was significant. This pattern is consistent with broader evidence that positive affect can sustain behavioral persistence even in the absence of elaborate strategy use (Feng and Hong, 2022; Kang and Wu, 2022; Li and Lerner, 2013; Reschly et al., 2008). Within the Chinese EFL ecology, this pathway may be especially relevant for maintaining routine practice (e.g., daily vocabulary memorization and listening drills), where confidence-derived enjoyment may help students persist through repetitive, monotonous preparation.

Third, the independent cognitive pathway (self-efficacy → CE → BE) was also significant. This reflects the forethought phase of self-regulated learning (Zimmerman, 2000), wherein learners translate confidence into deliberate strategy deployment, which then channels into behavioral investment (Mega et al., 2026; Teng, 2021). In the CET-4 preparation context, this pathway may characterize students who, despite anxiety about the exam format or limited intrinsic interest, rely on mastered test-taking strategies (e.g., timed reading drills, template-based writing practice, and structured error logging) to maintain consistent behavioral investment. Rather than being driven by enjoyment, their persistence may be steered by the belief that strategic effort will yield measurable score gains.

Together, these findings indicate that self-efficacy is associated with BE through multiple pathways in Chinese high-stakes EFL settings. Students may sustain active participation through positive affect, through deliberate strategy use, or through their combination. This flexibility in the belief-behavior translation highlights the adaptive nature of human agency: in a testing context where success depends on sustained behavioral investment, learners can draw on distinct psychological resources that may sustain effort associated with achievement.

### 5.3 The direct and indirect associations of self-efficacy with L2 achievement

The cumulative indirect effect through BE accounted for approximately 31% of the modeled total association between self-efficacy and L2 achievement. This suggests that a notable portion of self-efficacy’s association was carried by the hypothesized engagement pathway, in that efficacy beliefs are associated with EE and CE, which are also associated with behavioral investment and subsequent language performance. In the CET-4 preparation context, this indirect process is consistent with the routine accumulation of competence through sustained practice (e.g., vocabulary expansion, listening exercises, and periodic mock examinations), which gradually builds the linguistic resources required for the examination (Xie, 2013; S. Xu, 2021). In this sense, the hypothesized engagement pathway represents the long-term behavioral route through which self-efficacy is associated with achievement.

Alongside this indirect process, the direct effect of self-efficacy on L2 achievement was also significant, accounting for the larger share of the modeled total association. This suggests that efficacy beliefs may be associated with test performance through mechanisms beyond the hypothesized engagement pathway, warranting additional theoretical consideration. In high-stakes, time-constrained examinations (e.g., the CET-4), these mechanisms may include more adaptive coping with test-related stress or more efficient deployment of test-taking strategies throughout the preparation and examination period. For instance, students with strong efficacy beliefs may approach challenging listening materials with less anxiety during preparation and maintain more effective note-taking strategies in the actual examination (Canaran et al., 2020). Similarly, in the time-pressured reading section, confident students may allocate their time more adaptively, skipping difficult items strategically rather than panicking (Jiang, 2023). Unlike the indirect pathways that depend on months of sustained behavioral investment, this direct effect represents a statistically unmediated relationship with test performance. Because neither test anxiety nor strategy use during the test was directly measured, this interpretation remains speculative and awaits empirical verification.

Together, these findings suggest that self-efficacy is associated with L2 achievement through two theoretically distinct routes. One is indirect, operating through the hypothesized engagement pathway, consistent with the gradual accumulation of competence through sustained practice. The other is a direct association with test performance, which may reflect psychological resources that facilitate on-site adaptation under examination pressure.

Notably, while the direct effect was larger in magnitude than the cumulative indirect effect, the hypothesized engagement pathway nonetheless represents a significant and theoretically distinct indirect pattern. From an intervention perspective, the indirect pathway may be particularly consequential: whereas the direct association may partly reflect stable individual differences, the hypothesized engagement pathway involves malleable processes (EE, CE, and BE; Fredricks et al., 2004) that future instructional intervention studies could target.

### 5.4 Theoretical and practical implications

#### 5.4.1 Theoretical implications

This study addresses the methodological tendency to treat engagement sub-dimensions as interchangeable parallel mediators. By specifying a theory-guided sequential structure in which EE and CE are modeled as antecedents of BE, the findings show that these dimensions are distinct yet interconnected rather than competing predictors. This integrated framework advances the application of SCT in L2 acquisition by clarifying how internal psychological states may be linked to academic outcomes. Furthermore, the coexistence of significant direct and indirect associations suggests that self-efficacy is associated with achievement through distinct pathways, providing a more complete account of the belief-achievement link in high-stakes testing contexts.

#### 5.4.2 Practical implications

The identification of three pathways to BE suggests tentative directions for intervention research rather than direct instructional recommendations. First, because the independent affective pathway was significant, future studies could test whether fostering positive emotional experiences (e.g., interest-enhancing activities or low-anxiety classroom climates) increases routine behavioral investment. Second, because the independent cognitive pathway was also significant, intervention research could examine whether explicit strategy instruction supports students who maintain effort through deliberate self-regulation rather than intrinsic enjoyment. Third, because the sequential pathway from emotion to cognition was consistent with the data, future studies could examine interventions that combine confidence-building and strategic thinking. Finally, the significant direct association suggests that psychological preparation may be worth examining alongside content review—for instance, simulated practice under timed conditions, strategy-focused feedback on mock tests, or anxiety management techniques. All of these possibilities require experimental or longitudinal evaluation in future study.

### 5.5 Limitations and future directions

While the present findings advance understanding of how self-efficacy is associated with engagement dimensions in high-stakes EFL contexts, several limitations qualify the conclusions.

First, causal direction remains unresolved. Although the psychological variables were measured two months before the examination, self-efficacy, EE, CE, and BE were assessed concurrently: the design therefore separates the predictors from the outcome, but mediation among the T1 variables remains cross-sectional. The proposed ordering among the mediators was specified on theoretical grounds rather than empirically adjudicated (see Section 4.3.1). A related constraint is temporal granularity: engagement may fluctuate during the final review phase, when test-specific strategies and emotional states shift most dramatically, and the coping mechanisms underlying the direct self-efficacy–achievement association were not captured. Future research should employ multi-wave cross-lagged panel designs, more densely spaced measurements during the preparation phase, or quasi-experimental interventions to test directional claims and capture these dynamic processes.

Second, unmeasured confounders may inflate the observed associations. The models did not control for prior English proficiency, which may be associated with self-efficacy, engagement, and subsequent CET-4 performance alike; the reported associations may therefore partly reflect pre-existing differences in prior ability and cannot be attributed solely to the unique contribution of efficacy beliefs. No eligible baseline indicator was available in the present dataset, and future studies should incorporate objective pre-test scores, college entrance English scores, or freshman English grades to isolate the unique contribution of efficacy beliefs.

Third, key constructs were measured narrowly. L2 achievement was limited to a single standardized test score, which emphasizes receptive skills and may overlook productive competence. BE relied solely on self-report and thus captures perceived rather than observed behavioral engagement, and EE was measured as general affective engagement rather than momentary positive emotion. These features jointly limit the interpretation of BE as observed effort and the extent to which the findings directly test BBT. Although the adaptation followed the procedures described in Section 3.3, alternative factor structures were not compared. Future research should adopt multidimensional achievement assessments (e.g., oral interviews, writing portfolios), triangulate engagement with observational or digital trace methods, and consider experience-sampling measures of momentary emotion.

Despite these constraints, the present findings offer an integrative perspective on past inconsistencies in engagement research: the hypothesized sequential structure and the direct association between self-efficacy and L2 achievement provide both theoretical clarity and practical direction for high-stakes language learning contexts.

## 6 Conclusion

This study investigated how self-efficacy is associated with L2 achievement through the sub-dimensions of LE among Chinese EFL learners. The findings are consistent with a theory-guided sequential framework in which EE and CE were modeled as antecedents of BE, and BE was associated with subsequent academic outcomes. BE was the most proximal engagement dimension associated with internal psychological states and achievement; neither EE nor CE showed significant direct associations with L2 achievement when BE was accounted for. This pattern indicates statistical compatibility with the specified structure rather than an empirically established developmental chain.

Beyond this indirect process, self-efficacy also retained a significant direct association with L2 achievement. Together, these results indicate that efficacy beliefs are linked to test performance through two theoretically distinct routes: one involving indirect associations through sustained engagement in learning activities, and another involving a direct association through processes not fully captured by the measured engagement dimensions. While the direct association was larger in magnitude, the significant indirect pathways suggest that the hypothesized engagement pathway represents a theoretically distinct process.

These findings highlight that linking efficacy beliefs to language achievement in high-stakes contexts is not a uniform process. Learners may sustain behavioral investment through positive affect, through deliberate strategy use, or through their combination. Ultimately, effective language learning in this testing ecology may benefit from both the long-term behavioral accumulation supported by engagement and the direct self-regulatory resources for deploying competence under pressure.

## Data Availability

The raw data supporting the conclusions of this article will be made available by the authors, without undue reservation.
