# Supplementary material for: From belief to behavior: behavioral engagement as the proximal link between self-efficacy and L2 achievement
Source: Front Psychol. 2026 Oct 19;17:1997825. doi: 10.3389/fpsyg.2026.1997825 (PMC13724479; doi:10.3389/fpsyg.2026.1997825)
Supplement: Supplementary file 1 [file Table_1.docx]

| Table S1. Convergent and discriminant validity of the latent variables | | | | | | | |
| --- | --- | --- | --- | --- | --- | --- | --- |
|  | Item | Standardized loading | SMC | Error variance | CR | AVE | √AVE |
| Self-efficacy | SE1 | .759 | .576 | .424 | .901 | .566 | .752 |
|  | SE2 | .770 | .593 | .407 |  |  |  |
|  | SE3 | .821 | .674 | .326 |  |  |  |
|  | SE4 | .699 | .489 | .511 |  |  |  |
|  | SE5 | .756 | .572 | .428 |  |  |  |
|  | SE6 | .726 | .527 | .473 |  |  |  |
|  | SE7 | .728 | .530 | .470 |  |  |  |
| Behavioral engagement | BE1 | .630 | .397 | .603 | .885 | .494 | .703 |
|  | BE2 | .787 | .619 | .381 |  |  |  |
|  | BE3 | .786 | .618 | .382 |  |  |  |
|  | BE4 | .633 | .401 | .599 |  |  |  |
|  | BE5 | .677 | .458 | .542 |  |  |  |
|  | BE6 | .805 | .648 | .352 |  |  |  |
|  | BE7 | .676 | .457 | .543 |  |  |  |
|  | BE8 | .596 | .355 | .645 |  |  |  |
| Emotional engagement | EE1 | .706 | .498 | .502 | .916 | .579 | .761 |
|  | EE2 | .816 | .666 | .334 |  |  |  |
|  | EE3 | .816 | .666 | .334 |  |  |  |
|  | EE4 | .881 | .776 | .224 |  |  |  |
|  | EE5 | .808 | .653 | .347 |  |  |  |
|  | EE6 | .701 | .491 | .509 |  |  |  |
|  | EE7 | .709 | .503 | .497 |  |  |  |
|  | EE8 | .616 | .379 | .621 |  |  |  |
| Cognitive engagement | CE1 | .741 | .549 | .451 | .931 | .604 | .777 |
|  | CE2 | .782 | .612 | .388 |  |  |  |
|  | CE3 | .824 | .679 | .321 |  |  |  |
|  | CE4 | .828 | .686 | .314 |  |  |  |
|  | CE5 | .787 | .619 | .381 |  |  |  |
|  | CE6 | .849 | .721 | .279 |  |  |  |
|  | CE7 | .814 | .663 | .337 |  |  |  |
|  | CE8 | .761 | .579 | .421 |  |  |  |
|  | CE9 | .571 | .326 | .674 |  |  |  |

| Table S2. Structural model: unstandardized paths. | | | | | | |
| --- | --- | --- | --- | --- | --- | --- |
| Paths | *β* | S.E. | Est/SE | *p* | 95% CI | |
|  |  |  |  |  | LLCI | ULCI |
| SE→EE | .738 | .060 | 12.406 | < .001 | .622 | .855 |
| SE→CE | .173 | .048 | 3.629 | < .001 | .080 | .267 |
| EE→CE | .792 | .056 | 14.138 | < .001 | .682 | .902 |
| EE→BE | .277 | .067 | 4.11 | < .001 | .145 | .409 |
| CE→BE | .230 | .061 | 3.755 | < .001 | .110 | .350 |
| SE→BE | -.027 | .052 | -.527 | .598 | -.129 | .075 |
| BE→LA | .413 | .102 | 4.061 | < .001 | .213 | .612 |
| EE→LA | .081 | .141 | .575 | .565 | -.196 | .358 |
| CE→LA | .033 | .128 | .260 | .795 | -.218 | .285 |
| SE→LA | .279 | .111 | 2.515 | .012 | .062 | .496 |
| *Note:* SE = self-efficacy; BE = behavior engagement; EE = emotional engagement; CE = cognitive Engagement; LA = L2 achievement | | | | | | |

| Table S3. Structural model: standardized paths. | | | | | | |
| --- | --- | --- | --- | --- | --- | --- |
| Paths | *β* | S.E. | Est/SE | *p* | 95% CI | |
|  |  |  |  |  | LLCI | ULCI |
| SE→EE | .599 | .029 | 20.695 | < .001 | .542 | .656 |
| SE→CE | .132 | .036 | 3.694 | < .001 | .062 | .202 |
| EE→CE | .742 | .029 | 25.302 | < .001 | .685 | .800 |
| EE→BE | .323 | .075 | 4.29 | < .001 | .176 | .471 |
| CE→BE | .286 | .073 | 3.898 | < .001 | .142 | .431 |
| SE→BE | -.026 | .049 | -.527 | .598 | -.122 | .071 |
| BE→LA | .205 | .049 | 4.203 | < .001 | .109 | .300 |
| EE→LA | .047 | .082 | .575 | .565 | -.113 | .207 |
| CE→LA | .021 | .079 | .260 | .795 | -.135 | .176 |
| SE→LA | .131 | .052 | 2.536 | .011 | .030 | .232 |
| *Note:* SE = self-efficacy; BE = behavior engagement; EE = emotional engagement; CE = cognitive Engagement; LA = L2 achievement | | | | | | |

| Table S4. Sequential mediation pathways of self-efficacy to BE and L2 achievement (unstandardized coefficient) | | | | | | | |
| --- | --- | --- | --- | --- | --- | --- | --- |
| Paths | Mediating effects | Effect | S.E. | Est/SE | *p* | Bootstrap 95% CI | |
|  |  |  |  |  |  | LLCI | ULCI |
| SE-BE | Total Effect | .352 | .049 | 7.173 | < .001 | .256 | .448 |
|  | Direct Effect | -.027 | .052 | -.527 | .598 | -.129 | .075 |
|  | Indirect Effect | .379 | .044 | 8.552 | < .001 | .292 | .292 |
|  | Ind1: SE -EE -BE | .205 | .051 | 3.976 | < .001 | .104 | .305 |
|  | Ind2: SE -CE -BE | .040 | .015 | 2.599 | .009 | .010 | .070 |
|  | Ind3: SE -EE -CE -BE | .135 | .037 | 3.641 | < .001 | .062 | .207 |
| SE-LA | Total Effect | .509 | .087 | 5.822 | < .001 | .338 | .681 |
|  | Direct Effect | .279 | .111 | 2.515 | .012 | .062 | .496 |
|  | Indirect Effect | .230 | .070 | 3.295 | .001 | .050 | .345 |
|  | Inds through BE: |  |  |  |  |  |  |
|  | Ind4: SE-BE-LA | -.011 | .022 | -.522 | .602 | -.054 | .031 |
|  | Ind5: SE -EE -BE -LA | .084 | .029 | 2.897 | .004 | .027 | .142 |
|  | Ind6: SE -CE -BE -LA | .016 | .007 | 2.211 | .027 | .002 | .031 |
|  | Ind7: SE -EE -CE -BE -LA | .056 | .020 | 2.752 | .006 | .016 | .095 |
|  | Inds bypassing BE: |  |  |  |  |  |  |
|  | Ind8: SE -EE -LA | .060 | .104 | .575 | .565 | -.145 | .265 |
|  | Ind9: SE -CE -LA | .006 | .022 | .259 | .795 | -.038 | .049 |
|  | Ind10: SE -EE -CE -LA | .019 | .075 | .260 | .795 | -.128 | .167 |
| *Note:* Ind = indirect effect; SE = self-efficacy; BE = behavioral engagement; EE = emotional engagement; CE = cognitive Engagement; LA = L2 achievement; Bootstrap sample size = 5000; LLCI = low limit of conﬁdence; ULCI = upper limit of conﬁdence; | | | | | | | |

| Table S5. Self-efficacy scale (Adapted from Pintrich & De Groot, 1990) | | |
| --- | --- | --- |
| Item | Source | English /Chinese version |
| SE1 | Original Item 2 | I'm confident I can keep up with my classmates in English learning. 在英语学习上，我有信心跟上其他同学。 |
| SE2 | Original Item 7 | I'm certain I can understand the difficult material in English learning. 我确信自己能理解英语学习中的难点 |
| SE3 | Original Item 10 | I'm confident I can do very well in English learning. 我有信心在英语学习中表现得很好。 |
| SE4 | Original Item 11 | I'm confident I can be an outstanding English learner. 我有信心成为优秀的英语学习者。 |
| SE5 | Original Item 13 | I'm sure I can do an excellent job on English learning tasks. 我确信自己能出色完成英语学习任务。 |
| SE6 | Original Item 20 | I'm confident in my study skills for English learning. 我对自己的英语学习技能有信心。 |
| SE7 | Original Item 23 | I believe I can master what I learn in English. 我相信自己能够掌握英语学习的内容。 |
| *Note.* Original Items 15 and 22 were excluded; see Section 3.3 for the rationale; All items were administered in Chinese. | | |

| Table S6. Learning engagement scale (Adapted from Guo et al., 2022) | | |
| --- | --- | --- |
| Item | Source | English/Chinese version |
| BE1 | F8 (be12) | In English learning, I actively work with my partners to complete the assigned tasks. 在英语学习中，我积极与同伴合作完成布置的任务。 |
| BE2 | F8 (be14) | In English learning, I work hard to come up with novel ideas during group work. 在英语学习中，小组活动时我努力提出新颖的想法。 |
| BE3 | F6 (be2) | In English learning, I listen very carefully. 在英语学习中，我非常认真听讲。 |
| BE4 | F6 (be4) | In English learning, once my mind wanders, I make myself pay attention. 在英语学习中，一旦走神，我会让自己重新集中注意力。 |
| BE5 | F6 (be3) | In English learning, I easily get distracted. (-) 在英语学习中，我很容易分心。（反向题） |
| BE6 | F2 (be17) | I let the teacher know what I need and want in English learning. 在英语学习中，我会让老师了解我的需求和想法。 |
| BE7 | F8 (be15) | In English learning, I do just enough to get by during group work. (-) 在英语学习中，小组活动时我只求勉强应付。（反向题） |
| BE8 | F2 (be19) | In English learning, when I need some help, I ask the teacher for it. 在英语学习中，当我需要帮助时，我会向老师求助。 |
| EE1 | F3 (ee3) | English learning stimulates my curiosity. 英语学习能激发我的好奇心。 |
| EE2 | F1 (ee17) | I get deeply involved in the interactive activities in English learning. 我全身心投入英语学习中的互动活动。 |
| EE3 | F3 (ee1) | I think English learning is interesting. 我认为英语学习很有趣。 |
| EE4 | F3 (ee4) | English learning facilitates my imagination. 英语学习有助于发挥我的想象力。 |
| EE5 | F1 (ee19) | I feel happy communicating and interacting with my classmates in English learning. 在英语学习中，与同学交流互动让我感到快乐。 |
| EE6 | F1 (ee18) | I feel happy communicating and interacting with the teacher in English learning. 在英语学习中，与老师交流互动让我感到快乐。 |
| EE7 | F1 (ee15) | I like the interactions in English learning. 我喜欢英语学习中的互动。 |
| EE8 | F1 (ee14) | There is a good atmosphere for communication in English learning. 英语学习中有良好的交流氛围。 |
| CE1 | F4 (ce7) | In English learning, I memorize the newly learned words, phrases and sentence patterns by associating them with my own experiences. 在英语学习中，我会结合自身经历来记忆新学的单词、短语和句型。 |
| CE2 | F4 (ce3) | In English learning, I relate what I'm learning to what I already know. 在英语学习中，我会把正在学的内容与已有知识联系起来 |
| CE3 | F4 (ce15) | In English learning, I pay attention to the useful expressions used by my classmates and try using them in my own practice. 在英语学习中，我会关注同学使用的有用表达，并在自己的练习中运用。 |
| CE4 | F4 (ce14) | In English learning, I pay attention to the expressions used by the teacher and try using them in my own practice. 在英语学习中，我会注意老师使用的表达，并尝试在自己的练习中运用。 |
| CE5 | F5 (ce10) | In English learning, I actively ask the teacher questions so as to get more information during discussion activities. 在讨论活动中，我会主动向老师提问，以获取更多信息。 |
| CE6 | F5 (ce16) | In English learning, I ask the teacher questions when I don't understand something clearly. 在英语学习中，遇到不清楚的地方，我会向老师提问。 |
| CE7 | F5 (ce12) | In English learning, I talk with the teacher to help plan how to improve my English. 在英语学习中，我会与老师交流，帮助自己规划如何提高英语水平。 |
| CE8 | F7 (ce11) | In English discussion, I actively ask my classmates questions so as to get more information. 在英语讨论活动中，我会主动向同学提问，以获取更多信息 |
| CE9 | F7 (ce17) | In English discussion, I briefly summarize and take notes of the different useful ideas put forward by my classmates. 在英语讨论活动中，我会简要总结并记录同学提出的不同观点。 |
| *Note.* 25 items were adapted from Guo et al. (2022) ; see Section 3.3 for the rationale; All items were administered in Chinese; Item BE5 and BE7 was reverse-scored. | | |
